# A mid-infrared focusing grating coupler with a single circular arc element based on germanium on silicon

**DOI:** 10.3762/bjnano.14.38

**Published:** 2023-04-06

**Authors:** Xiaojun Zhu, Shuai Li, Ang Sun, Yongquan Pan, Wen Liu, Yue Wu, Guoan Zhang, Yuechun Shi

**Affiliations:** 1 School of Information Science and Technology, Nantong University, Nantong 226019, China; 2 Yongjiang Laboratory, Ningbo 315202, China and Nanjing University, Key Lab Intelligent Opt Sensing & Manipulat, Minist Educ, Nanjing University, Nanjing 210093, China

**Keywords:** circular arc element, focusing grating coupler, germanium-on-silicon, mid-infrared

## Abstract

A mid-infrared (MIR) focusing grating coupler (FGC) with a single circular arc element (CAE) in the front of the gratings based on a germanium-on-silicon (Ge-on-Si) platform is designed and demonstrated. It can be used equivalently to a traditional FGC with all-focusing gratings. By optimizing the structural parameters of the CAE, the combination of a tapered linear grating and the CAE can improve the coupling efficiency to 8.61%, which is twice as large as that of the traditional MIR grating couplers. To the best of our knowledge, it is the highest coupling efficiency in a full-etch grating coupler based on Ge-on-Si. Moreover, the proposed grating coupler can be used for refractive index (RI) sensing, and the maximum sensitivity is 980.7 nm/RIU when the RI changes from 1 to 1.04. By comparing with traditional grating couplers requiring secondary etching, the proposed full-etch grating coupler structure can reduce the complexity of fabrication and can provide a prospective platform for MIR photonic integration and photonic biosensor detection.

## Introduction

The mid-infrared (MIR) spectrum region covers the absorption band of most organic and inorganic matter. Thus, it has a broad application prospect in gas detection, environmental monitoring, lidar, free space optical communication, and remote sensing technologies [1–2]. The recombination of chemical bonds caused by changes in molecular structures can induce significant differences in MIR spectra. Thus, slight differences in the structure of compounds or molecules (such as isomers) can be distinguished by mid-infrared spectroscopy [3]. Therefore, this spectral region is called “fingerprint spectrum region” (FSR) [1,4]. Many small biological molecules have unique and identifiable absorption spectra in the MIR band of 6–15 μm [1,5]. It is of great application value to develop photonic biosensors in this FSR. The spectral transparency window of germanium can fully cover the wavelength of 6–15 μm. Hence, it is a suitable material for biosensors applications in the MIR band [6].

In recent years, researchers have verified the feasibility of Ge MIR waveguides on various substrate materials, such as germanium on silicon (Ge-on-Si), germanium on silicon-on-insulator (GOSI) [7], germanium on insulating substrate (GOI) [8–9], and germanium on silicon nitride substrate (GOSN) [10]. Among them, Ge-on-Si platforms have been widely applied in on-chip sensors, nonlinear optics, free space communication, and thermal imaging [1,6] because portable, cost-effective, and mass-produced integrated systems can be made from such platforms [6]. One key technology is how to couple the MIR light efficiently into the Ge-based waveguides. Many MIR grating couplers have been proposed and demonstrated to achieve this purpose [1,11]. In 2016, Alonso-Ramos et al. reported a Ge-on-Si grating coupler with an inverse taper excitation, operating near 3.8 μm wavelength with a maximum coupling efficiency of −11 dB (7.9%) [11]. In 2017, Kang et al. designed and experimentally demonstrated a focusing subwavelength grating (SWG) for an efficient coupling of MIR light to a suspended membrane Ge waveguide [1]. The maximum coupling efficiency was −11 dB at the focusing SWG’s center wavelength of 2.37 μm. The high coupling efficiency was obtained experimentally. However, the fabrication flow of the suspended membrane Ge waveguide with focusing SWGs greatly increased the complexity of the technological process. Therefore, a focusing grating coupler (FGC) with a simple fabrication process and high coupling efficiency is urgently needed.

In this paper, a MIR FGC with a single circular arc element (CAE) based on Ge-on-Si is demonstrated and simulated. The proposed MIR FGC consists of a section of tapered linear gratings and a single CAE, which can be equivalent to the traditional FGC with all-focusing gratings. Also, it is a full-etch grating coupler, which can be achieved by a single etch step. The maximum coupling efficiency can be up to 8.61% (−10.65 dB) at 6.878 μm by optimizing the structural geometry of the CAE. To the best of our knowledge, it is the highest coupling efficiency in full-etch grating couplers based on Ge-on-Si. Moreover, the proposed MIR FGC can also be used for sensing, and the maximum refractive index (RI) sensitivity is 980.7 nm/RIU. Compared with the suspended membrane Ge waveguide with a focusing subwavelength grating MIR grating coupler, the difficulty of preparation has been considerably reduced.

## Principle and Design

**Figure 1:**
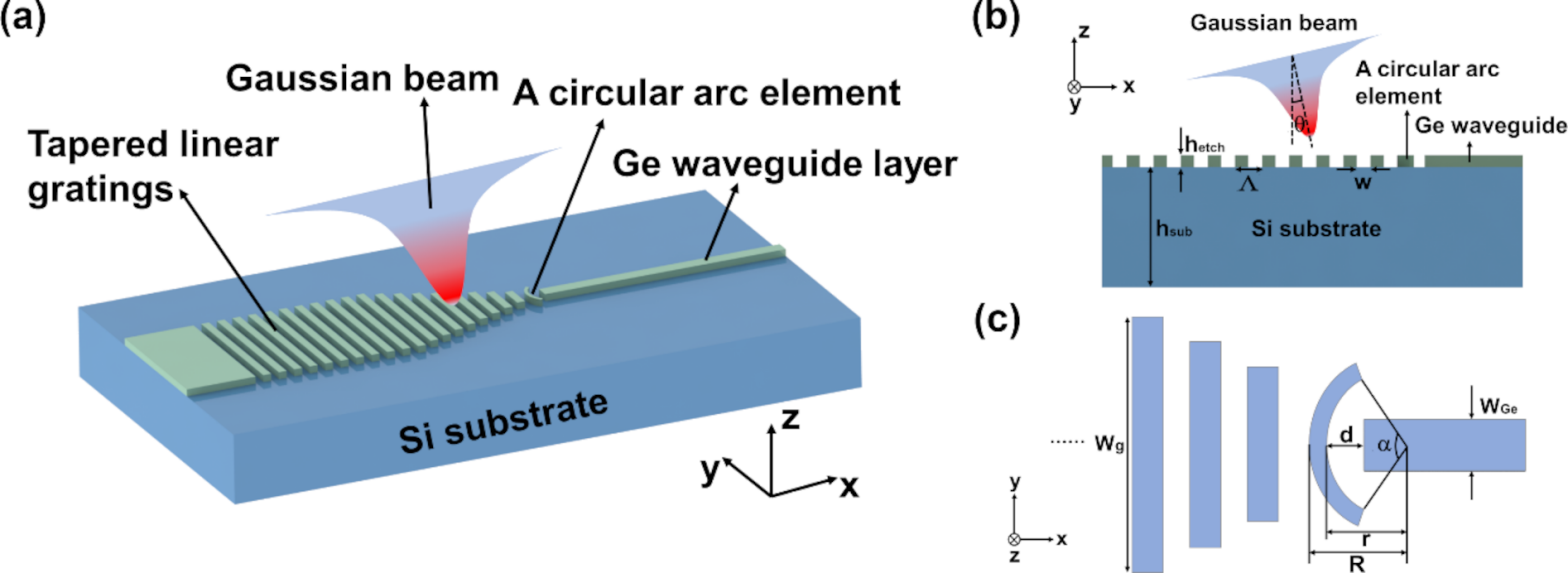
(a) Tilted view and (b) cross-sectional view of the proposed MIR grating coupler. (c) Schematic diagram of the proposed CAE in the MIR grating coupler.

Figure 1a shows the tilted view of the proposed MIR FGC. The Ge waveguide layer is built onto the Si substrate forming the Ge-on-Si structure. The proposed MIR FGC consists of a section of tapered linear gratings and a single CAE. Figure 1b shows the cross-sectional view of the proposed MIR FGC. The grating period is Λ, the width of the trenches is *w*, and the duty cycle is defined as *f* = *w*/Λ. The Ge waveguide thickness is *h*_etch_, which is also the etching depth. The incident angle is θ. In our work, the numerical simulations have been performed by using a commercial software of Lumerical FDTD solutions, which is based on the finite-difference time-domain method, and the light source we used for exciting the grating coupler is a Gaussian laser beam. The details of the Gaussian beam are as follows: The injection axis is the *z* axis, the waist radius is 3 µm, and the center wavelength is 7 μm.

The coupling mechanism of the grating can be characterized as [11]:


[1] $$n_{1}\sin\left(\theta \right)=n_{\text{eff}}+\frac{k\lambda }{\Lambda },$$

where *n*_1_ is the refractive index of the surrounding air (*n*_1_ = 1), θ is the coupling angle, λ is the wavelength of the incident light in vacuum, *n*_eff_ is the effective refractive index of the fundamental mode in the grating, and *k* is the diffraction order. By convention, the period of the grating couplers is small enough to guarantee that only the first diffraction order (*k* = −1) satisfies the diffraction condition to produce single-beam diffraction [11]. However, the Ge thickness of Ge-on-Si grating couplers is generally about 2 μm. In addition, if the grating is designed for first-order diffraction (*k* = −1), it will lead to gratings with a high aspect ratio (defined as the ratio between the etch depth and the width of the grating trench). All these factors increase the difficulty of fabrication in practice. Therefore, Ge-on-Si grating couplers should be designed to work with the second-order diffraction (*k* = −2) [11].

Figure 1c is the schematic diagram of the proposed CAE in the MIR FGC. The CAE is located between the tapered linear gratings and the output Ge waveguide. *R* and *r* are the outer and inner radii of the CAE, respectively. *d* represents the position of the CAE, and α is the opening angle of the CAE. *w*_g_ is the width of the grating in the *y* direction, which is scanned from 40 to 12 µm in interval steps of 4 μm in the simulation. *w*_Ge_ is the width of the output Ge waveguide, which is set to 4 μm. The CAE we designed is used to replace a linear grating at the front of the tapered gratings. The focusing effect of the CAE is conducive to coupling the light incident on the surface of the grating coupler to the narrow waveguide. The CAE can also help to reduce reflection and to couple more light into the narrower waveguide to be transmitted forward. In addition, the proposed MIR FGC can be used as a spot-size converter while coupling light from a fiber into the Ge waveguide because of the small size in comparison with a conventional inverted taper grating coupler [12]. Therefore, the combination of the tapered linear gratings and the CAE is not only beneficial to decrease the size of the grating coupler. It also strongly increases the coupling efficiency of the MIR grating coupler.

## Results and Discussion

In our work, the incident light angle and the shape of the CAE are the main adjustable parameters when studying the coupling efficiency. The FGC is a full-etch grating with a grating period Λ of 4.5 μm. The Ge waveguide thickness *h*_etch_ is set to 2 μm, the duty cycle *f* is 0.5, and *k* = −2.

**Figure 2:**
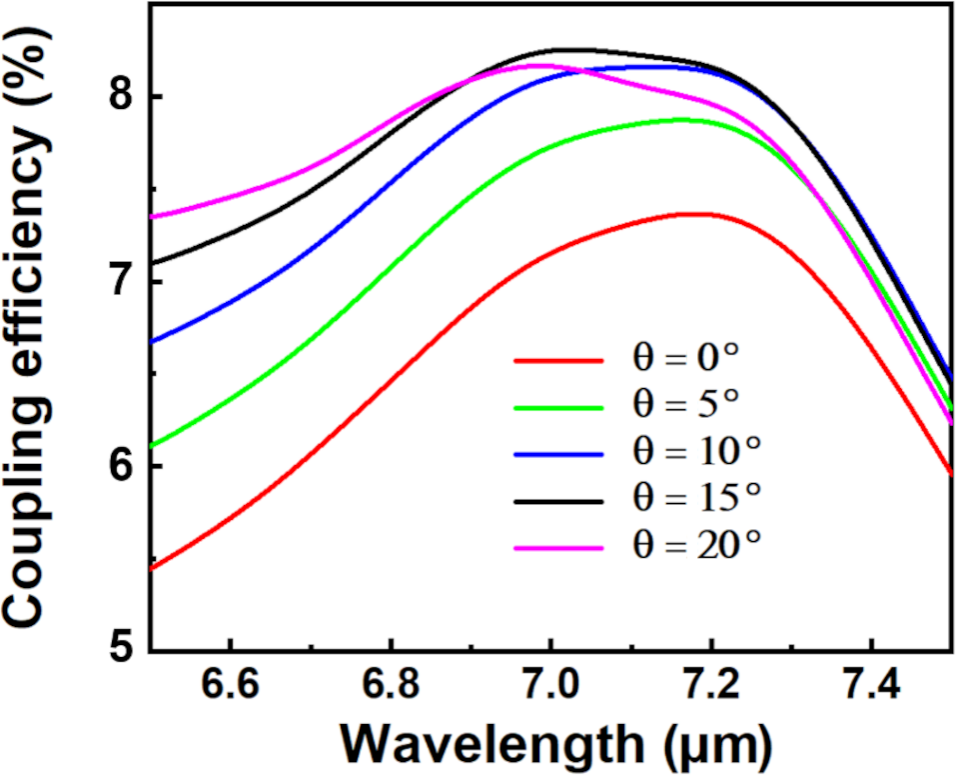
Coupling efficiency of the MIR FGC at different incident angles when *R* = 7.15 μm, *r* = 6.15 μm, *d* = 3.55 μm, and α = 120°.

Figure 2 shows how the incident angle θ impacts the coupling efficiency when *R* = 7.15 μm, *r* = 6.15 μm, *d* = 3.55 μm, and α = 120°. It can be seen that with the increase of θ, the coupling efficiency first increases, then reaches the maximum value of 8.25% (7.027 μm) when θ = 15°, and finally decreases again. Therefore, an incident angle of θ = 15° yields more light coupled into the Ge waveguide. It should be noted that all coupling efficiencies are above 7% for θ values of 0°–20°, which can be attributed to the fact that the designed MIR grating structure fulfils the Bragg diffraction condition of Equation 1 very well.

**Figure 3:**
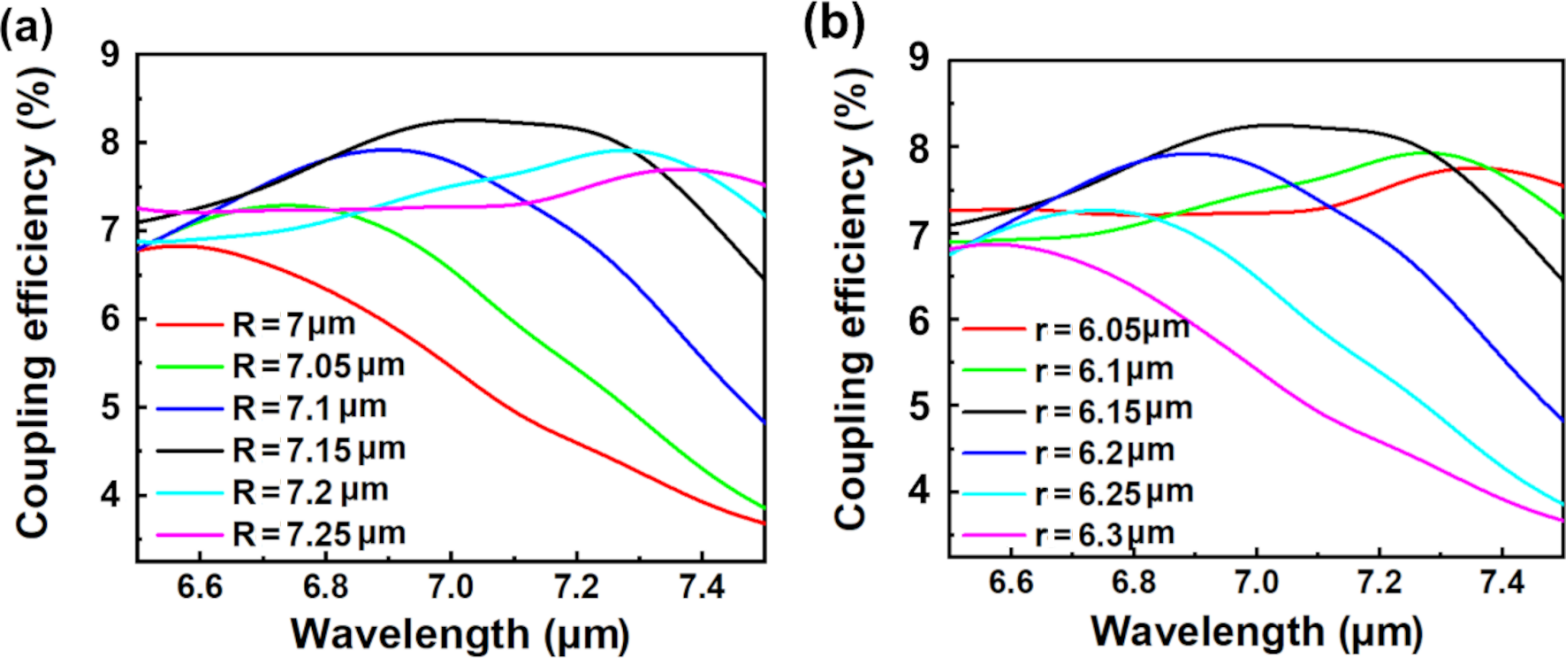
Coupling efficiency of the proposed MIR FGC at different (a) outer radii (*r* = 6.15 μm) and (b) inner radii (*R* = 7.15 μm) with θ = 15°, *d* = 3.55 μm, and α = 120°.

It is also interesting to see the impacts of the different inner and outer radii of the CAE on the coupling efficiency. The designed parameters are set as θ = 15°, *d* = 3.55 μm, and α = 120°. Figure 3a shows the coupling efficiency at different *R* when *r* is fixed to 6.15 μm. The maximum coupling efficiency of 8.25% (7.027 μm) is obtained when *R* is 7.15 μm. Figure 3b shows the coupling efficiency at different *r* when *R* is fixed to 7.15 μm. The maximum coupling efficiency is 8.25% (7.027 μm) when *r* is 6.15 μm. It can be seen that the coupling efficiency is almost equal when the values of *R* and *r* are changed separately. This is because the changing values of *R* or *r* are equivalent to the coupling efficiency with fixed incident angle and position of the CAE.

**Figure 4:**
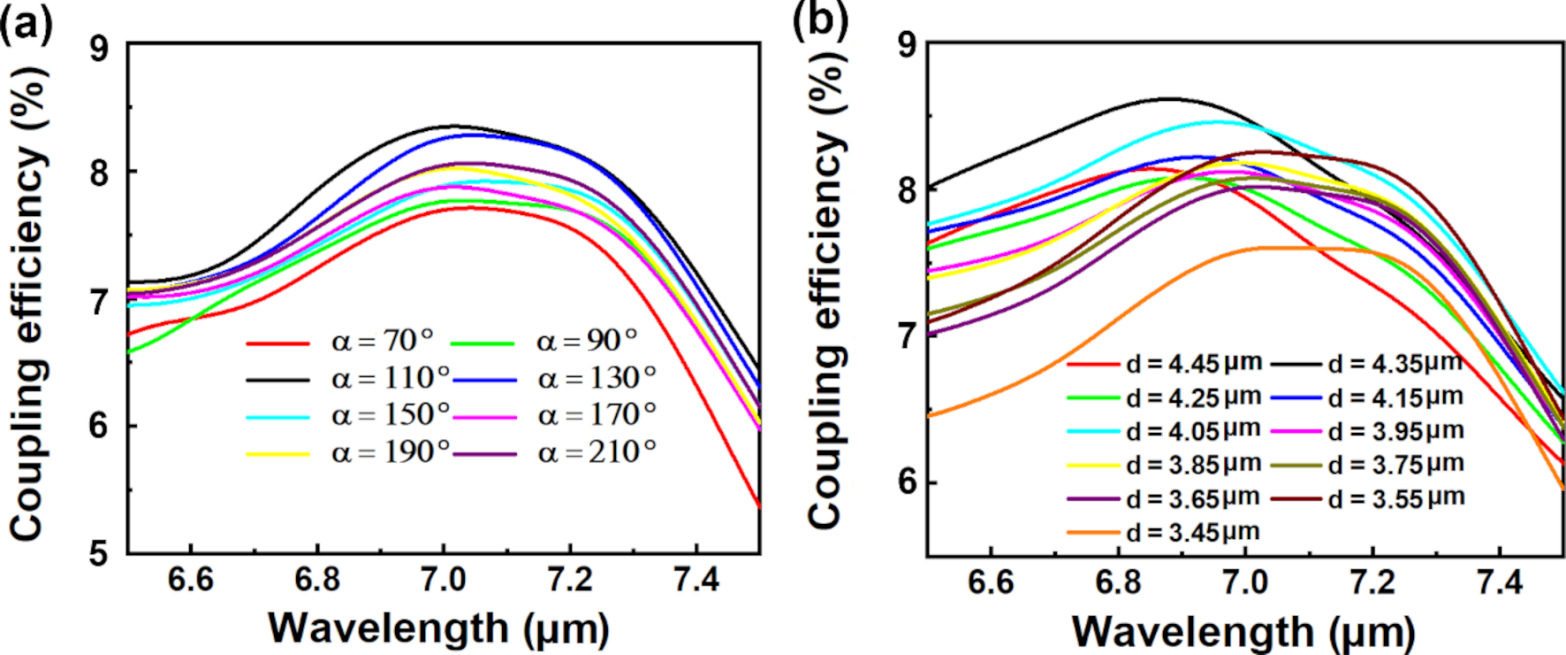
Coupling efficiency of the proposed MIR FGC at different (a) opening angle (*d* = 3.55 μm) and (b) location (α = 120°) with θ = 15°, *R* = 7.15 μm, and *r* = 6.15 μm.

Figure 4 shows the effects of the CAE opening angle α and position *d* on the coupling efficiency of the proposed MIR FGC using the parameters θ = 15°, *R* = 7.15 μm, and *r* = 6.15 μm. The coupling efficiency changes with the change of α and reaches the maximum of 8.35% (7.017 μm) when α = 110°, as shown in Figure 4a. Figure 4b shows that the parameter *d* can also affect the coupling efficiency of the proposed MIR FGC, and the maximum coupling efficiency is 8.61% (6.878 μm) when *d* = 4.35 μm. Furthermore, the total insertion loss (IL) has been estimated, which can be expressed as [13]:


[2]

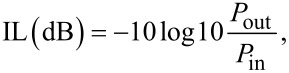



where *P*_in_ and *P*_out_ stand for input power and output power, respectively. Under the condition of maximum coupling efficiency, the value of *P*_out_/*P*_in_ is around 0.647 obtained from simulation. Thus, an IL value of around 8 dB has been calculated using Equation 2.

**Figure 5:**
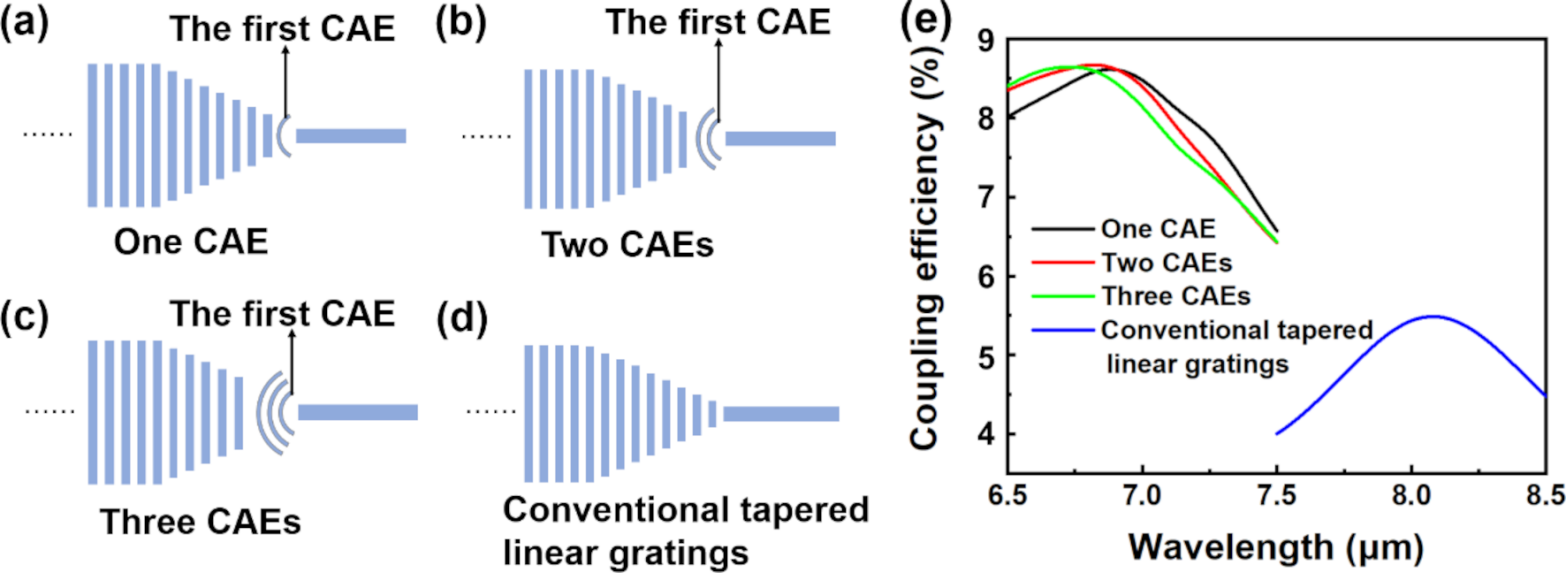
Structures of MIR grating couplers with (a) one single CAE, (b) two CAEs, (c) three CAEs, and (d) conventional tapered linear gratings. (e) Comparison of coupling efficiency of MIR FGCs with different structures.

In order to highlight the advantages of our proposed MIR FGC with a CAE, we have simulated the coupling efficiency of MIR grating couplers with different numbers of CAE and conventional tapered linear gratings, as shown in Figure 5. The simulation conditions are Λ = 4.5 μm, *f* = 0.5, θ = 15°, and *h*_etch_ = 2 μm. *d* of the first CAE is 4.35 μm, α of the CAEs is 120°, and period and duty cycle of the CAEs are 4.25 μm and 0.76, respectively. Comparing the coupling efficiency of MIR FGCs with one CAE, two CAEs, and three CAEs, we can see that the coupling efficiencies are nearly equal, all reaching 8.6%, as shown in Figure 5e. This fact indicates that the coupling efficiency achieved by a single CAE is almost equal to that obtained with multiple CAEs (i.e., with traditionally focusing gratings). This is very significant regarding the practical manufacturing with the requirements of reducing production cost and complexity while pursuing high coupling efficiency. Moreover, it should be emphasized that the coupling efficiency of the proposed MIR FGC with a single CAE (8.61%) is much higher than that of a MIR grating coupler with conventional tapered linear gratings (5.49%). Because the proposed structure is geometrically simple, it is easy to implement experimentally. The proposed MIR FGC based on Ge-on-Si can be fabricated by electron beam lithography (EBL) and inductively coupled plasma etching (ICP). EBL is used to produce lithographically the grating pattern into the resist, which can be further transferred onto the Ge layer by ICP. This is a simple manufacturing process that requires only one single etch step. Then, we can use a continuous-wave single-frequency tunable MIR laser (the center wavelength is ca. 7 μm) as the light source to test the coupling efficiency of the experimental samples. The MIR laser is coupled into a single-mode ZrF_4_ optical fiber via a black diamond-2 aspheric lens [1]. The light from the single-mode ZrF_4_ optical fiber is coupled into the proposed MIR grating coupler [14]. Finally, the transmission characteristics of the output fiber can be detected using an optical spectrum analyzer.

**Figure 6:**
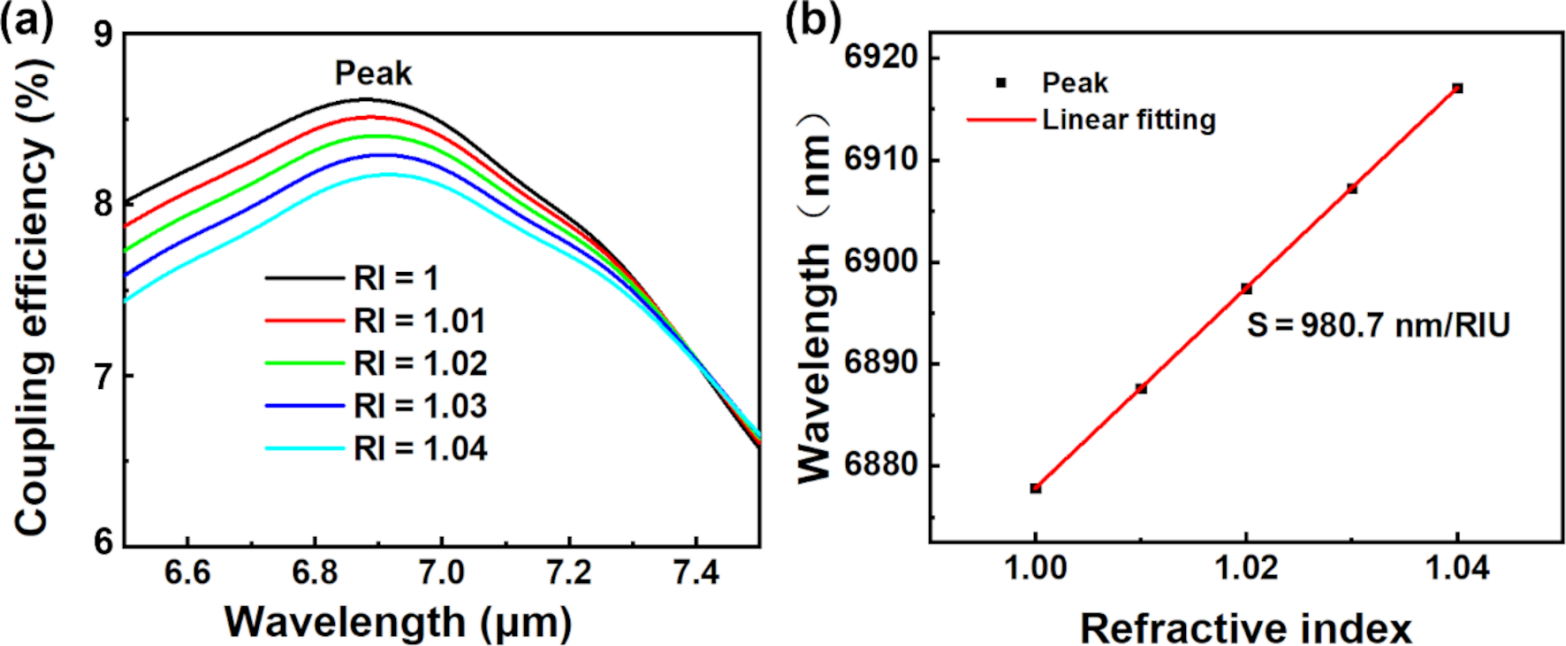
Sensing performance of the proposed MIR FGC.

There is a wide range of sensors for applications in, for example, biosensing, healthcare, disease detection, and gas detection. Therefore, research on those sensors is of great significance. In 2015, Bai et al. reported a flexible healable transparent chemical gas sensing device that exhibited robust flexibility, good transparency, and reliable water-enabled healability of the gas sensing performance at room temperature [15]. Wang proposed a flexible, transparent, and portable wrist strap sensor and a mechano-based transductive sensor in 2017 and 2018, respectively [16–17]. They have good application prospects in healthcare. In 2020, Xue et al. reported a bismuthene-enabled fluorescence quenching biosensor to detect microRNA, which is relevant to the fields of biosensors and medicine [18]. In 2022, Chen et al. demonstrated a methodology of photonic clustered regularly interspaced short palindromic repeat (CRISPR) sensing for rapid and specific diagnosis of the Omicron variant of SARS-CoV-2 [19]. This innovative CRISPR-empowered surface plasmon resonance platform will further contribute to the field of biomedical sensors. We also studied the sensing performance of our proposed MIR FGC when it worked as a sensor rather than a coupler, as shown in Figure 6. When RI increases from 1 to 1.04, the peak of coupling efficiency shows a redshift from 6877.8 nm to 6917.1 nm. The RI sensitivity is 980.7 nm/RIU obtained from a linear fit of the peak wavelength and RI, as shown in Figure 6b. The sensitivity is twice as large as that in [20]. Furthermore, the proposed sensor is a full-etch structure based on Ge-on-Si, which can be achieved by a single etch step. The manufacture is simple compared with multiple etching [1]. Therefore, after considering the production process, production cost and sensitivity, our proposed sensor based on Ge-on-Si is expected to have commercially available applications in the future.

## Conclusion

In summary, we designed and demonstrated a full-etch MIR FGC with a single CAE. The coupling efficiency could be tuned by changing the structural parameters of the CAE and the incident angle of light. The maximum coupling efficiency of 8.61% (−10.65 dB) was obtained at a wavelength of 6.878 μm. Moreover, the coupling efficiency of the single CAE was equivalent to that of multiple CAEs, such as in focusing gratings, which could significantly reduce the production cost and complexity while keeping high coupling efficiency. In addition, the RI sensing performance of the proposed MIR grating coupler was also simulated when it was used as a sensor. The RI sensitivity of the sensor was 980.7 nm/RIU. Therefore, the proposed MIR FGC would provide a potential platform for MIR photonic integration and photonic biosensors detection based on chips.
